# ‘Oh! It's Like Taboo’: Perspectives of Religious and Community Leaders on AOD Use, Harms and Treatment in CALD Communities, Sydney

**DOI:** 10.1002/hpja.70105

**Published:** 2025-09-23

**Authors:** Winifred Asare‐Doku, Robert Stirling, Prince Peprah, Catherine Foley, Nirekha De Silva, Teguh Syahbahar, David Kelly, Stella Settumba

**Affiliations:** ^1^ National Drug and Alcohol Research Centre University of New South Wales Sydney Australia; ^2^ Drug Policy Modelling Program, Social Policy Research Centre, UNSW Sydney Australia; ^3^ The Network of Alcohol and Other Drugs Agencies Sydney Australia; ^4^ Australian Institute of Health Innovation, Macquarie University Sydney Australia; ^5^ Odyssey House NSW Sydney Australia

**Keywords:** alcohol, community leaders, drugs, harms, multicultural, religious leaders, treatment

## Abstract

**Objective:**

Alcohol and other drug (AOD) use is a growing social and health issue, and culturally appropriate treatment is important, especially for people from culturally and linguistically diverse (CALD) backgrounds. This study aimed to explore the views of religious and community leaders on AOD use and treatment in CALD communities in New South Wales, Australia.

**Design:**

Using a qualitative exploratory approach, eight religious and community leaders were purposively selected from Sub‐Saharan African, North African, Middle Eastern, East Asian and Pacific Islander communities. This approach is particularly suited to contexts where little prior research exists, allowing for in‐depth insights into participants' views.

**Results:**

The study identified four key themes in participants' perspectives on AOD use and treatment within CALD communities. First, cultural and contextual factors described how cultural and religious values influence community leaders' attitudes toward AOD. Second, community‐wide impact captured the collective nature of CALD communities, where individual behaviours are seen as reflecting on the broader family and community. Third, enablers to access and engagement with AOD Services highlighted the factors that support access to and engagement with AOD services. Finally, barriers to access and engagement with AOD Services addressed the obstacles that prevent individuals from seeking treatment.

**Conclusion:**

Our findings show that AOD use and harms can be influenced by contextual and cultural factors, requiring culturally appropriate and targeted interventions and strategies to prevent harm and to manage AOD treatment among CALD communities.

**Implications for Public Health:**

Findings highlight the need for culturally tailored AOD prevention and treatment strategies that engage community and religious leaders to improve access, reduce stigma and increase uptake within CALD communities.

## 1 Introduction

Nearly 52% of the Australian population is classified as having a culturally and linguistically diverse (CALD) background [1]. The definition of CALD is being born overseas or having one or both parents born in non‐English speaking countries [1]. The people who make up these unique CALD communities have diverse languages, ethnic backgrounds, nationalities, traditions, societal structures and religions [1]. Some people of CALD background have also experienced historical trauma, grief, loss, structural and socio‐economic barriers which make them more susceptible to developing risky coping strategies [2, 3, 4, 5]. An increasing concern for CALD communities is alcohol and other drug (AOD) use, with harmful patterns of drinking and drug use among some groups of CALD background being reported [6, 7]. Additionally, there is limited access to culturally sensitive AOD programme and treatment services [8, 9].

Despite the documented prevalence of AOD problems, however, people with CALD backgrounds are still underrepresented in AOD treatment settings [3, 9]. Consequently, exploring the barriers to seeking AOD support services and the unique needs of people who have CALD backgrounds is vital to reducing the harms in CALD communities [1]. The National Framework for alcohol, tobacco and other drug treatment guides a national response on treatment interventions [10]. It comprises three key types of interventions: harm reduction strategies; screening, assessment and care coordination; and intensive treatment [10]. These components collectively aim to reduce AOD‐related harms, support early identification and engagement and provide targeted care for individuals. In New South Wales (NSW), AOD services are delivered through a comprehensive system coordinated by NSW Health [11]. These services are provided by both Local Health Districts (60%) and non‐government organisations (40%), and include a range of treatment and support options, such as counselling, withdrawal management and residential rehabilitation [12]. Over the past decade, counselling has consistently been the most common treatment type [12].

Stigma is a well‐documented barrier to seeking treatment for AOD problems [13, 14]. Stigma is a phenomenon in which individuals are judged or disapproved of by others because of a particular trait or characteristic they possess. In CALD communities, key contributors to stigma can arise from deeply ingrained cultural and religious values that shape community attitudes toward AOD use [14]. A recent systematic review, for example, highlighted that family members and leaders in CALD communities often view people who use illicit drugs as undeserving of dignity or respect [14]. These individuals also face rejection from their friends, intensifying the detrimental effects of stigma. Concerns about privacy and exposure reportedly deter many people of CALD backgrounds from seeking treatment, particularly in services staffed by members of their own community or those with long wait times, where their presence might become noticeable to others [14]. Although stigma continues to be reinforced by cultural and religious norms in CALD communities, help‐seeking for AOD problems is negatively impacted [15].

Given this central role that cultural and religious values play in shaping behaviours within CALD communities, religious and community leaders often act as influential gatekeepers, guiding their communities' attitudes and responses to issues such as AOD use and access to treatment [16]. Many CALD communities tend to be less trusting of the health system and government institutions, often turning instead to their religious and community leaders because the leaders live within the community, are known to them, are regarded as trusted authorities and the first point of contact in times of crisis [17, 18]. One study found that religious leaders in the African community significantly influenced the health behaviours of community members through scriptural guidance, social influence and role modelling, particularly regarding alcohol and tobacco use [19]. Other studies report the value of collaboration with community and religious leaders in addressing various social issues including natural disasters [18], the COVID pandemic [20], suicide prevention [21] and family violence [16]. Research continues to highlight the critical role that religious and community leaders play in shaping attitudes toward health behaviours, particularly in minority and migrant communities.

Understanding the perspectives of religious and community leaders is therefore essential for enhancing outreach, support and treatment services in CALD communities [22]. Collaborating with these leaders could also assist in the co‐development of culturally appropriate interventions that resonate with community values and beliefs, ultimately leading to more effective health promotion strategies [22]. Their contribution to designing and implementing culturally safe AOD interventions is central to building trust and improving health outcomes in CALD populations [23]. There are limited studies, however, examining the perspectives of these leaders in CALD communities in Australia.

We sought critical insights into how health interventions and policies are received and implemented at the grassroots level as well as potential clinical implications. By gaining insight from the people who hold influential support roles in CALD communities, the factors that either facilitate or hinder individuals' willingness to seek help can be better understood. Further research is needed to explore the cultural and social mechanisms influencing AOD use and treatment outcomes in CALD communities. This includes investigating how traditional treatment approaches can be adapted to better align with cultural sensitivities and engaging religious and community leaders to inform more culturally responsive policy, practice and programme design. The current study therefore aims to understand the perceptions of religious and cultural leaders toward AOD use, harms and treatment access in CALD communities in NSW.

## 2 Materials and Methods

### 2.1 Setting and Participants

The study used a qualitative approach. Participants (*n* = 8) were purposively recruited from predominant multicultural groups in Southwest and Western Sydney, Australia. NSW, one of Australia's most culturally diverse states, has a substantial population from CALD backgrounds. These communities, particularly those with African, Asian and Pacific Islander heritage, are predominantly located in regions such as South‐Western, Inner West and Western Sydney [24, 25]. Participants were eligible to take part in the study if they lived in Western or South‐Western Sydney, identified as being of Sub‐Saharan African, North African, Arabic‐speaking Asian or Pacific Islander descent, were able to communicate in English, and held a recognised role as a community leader or elder within their community. Four religious and four community leaders were recruited from these communities. The cultural background of participants included Sub‐Saharan African, North African and Middle Eastern, East Asian and Pacific Islander descent. The project team worked with a specialised multicultural organisation in Western Sydney to help build trust with the leaders and identify participants. Participants were then contacted via email, phone, or face‐to‐face with information about the research. Interested participants were then recruited.

### 2.2 Recruitment and Data Collection

Ethical approval for the study was from the University of New South Wales Human Research Ethics Committee (iRECS0846). Recruitment was conducted through email dissemination across networks of community and religious leaders associated with the targeted CALD communities, as well as through word‐of‐mouth referrals. One of the study authors (T.S.), who holds a leadership role within the community, further supported recruitment by engaging with his own contacts within these communities. Interested individuals who met the eligibility criteria responded via email or telephone and were subsequently invited to participate in interviews, which were conducted in person, via video conferencing or over the phone, according to their preference. The interviewers are both clinicians and researchers of a CALD background who speak English as a second or third language and who live within the CALD communities [16]. Prior to all the interviews, potential participants were provided with verbal and written information about the study and their consent sought to be interviewed and audio recorded. Data collection was between November 2023 and December 2023, and a semi‐structured interview guide was used. The interview guide focused on their beliefs and perceptions about AOD use, as well as treatment enablers and barriers within the communities. At the start of the interview, participants were asked to complete a short demographic questionnaire including characteristics such as gender, age range, cultural background and country of birth. Interviews were conducted both in person at mutually convenient locations or via a secured Zoom platform, and they lasted between 40 and 80 min. Participants were provided with $50 grocery vouchers as acknowledgement for their contribution to the project.

### 2.3 Data Analysis

A professional transcriber transcribed the interviews after signing a non‐disclosure agreement. Interviews were transcribed verbatim and checked for accuracy against audio recordings. Data was stored and coded in QSR NVivo (v1.3). A thematic analysis framework [26] to analyse the interview transcripts was used. Two authors coded the data. The coders familiarised themselves with the data and identified recurring patterns and keywords in the data. Initial inductive coding was then completed by assigning codes to phrases and words within the data, being guided by the objectives of the study while remaining open to emergent patterns and meanings beyond the interview guide. This approach allowed for the identification of themes that reflected both the participants' lived experiences and broader thematic connections [27]. Codes were compared and refined through discussion to ensure consistency and enhance reliability. The codes were systematically sorted and grouped into categories to form themes [26]. Some initial codes were categorised and developed into themes and subthemes. Data were recoded as additional themes were derived. All themes were further reviewed to ensure they captured the study objectives.

## 3 Results

Table 1 shows demographic characteristics of the participants. Of the eight participants, six were born in non‐English speaking countries, with two born in Australia and New Zealand. Participants commonly spoke English in addition to their ethnic language at home. The ethnicities reflect the diverse groups in Western and Southwestern Sydney. Seven participants were employed full‐time and also fulfilling their roles as leaders in the community.

**TABLE 1:** Demographics of study participants (*N* = 8).

Demographic categories	*N*
Gender	
Male	6
Female	2
Age	
25–29	1
40–44	3
45–49	1
55–59	1
65+	2
Ethnicity	
Sub‐Saharan African	2
North African/Middle Eastern	2
East Asian	2
Pacific Islanders	2
Religion	
Islam	2
Christian	5
Buddhism	1
Role in the community	
Community leader	4
Religious leader	4

## 4 Themes

Four major themes with seven sub‐themes were derived from the data that described perceptions about AOD use and treatment enablers and barriers (Table 2). For purposes of anonymity, quotes are attributed to participants in the following format: (role in the community [religious or community leader], participant number).

**TABLE 2:** Summary of themes and sub‐themes.

Themes	Sub‐themes
Cultural and contextual factors	Cultural perceptions and values; social dynamics and access
Community‐wide impact	
Treatment enablers	Culturally responsive approaches; involving families; safe and supportive environments
Barriers to access and engagement with AOD services	Stigma and shame; communication and awareness; cultural competence

### 4.1 Theme 1: Cultural and Contextual Factors

This theme reflects how cultural and religious norms, beliefs and values shape the attitudes and perceptions of the leaders toward AOD within CALD communities. Under this theme, two sub‐themes were derived: cultural perceptions and values, and social dynamics and access.

#### 4.1.1 Cultural Perceptions and Values

There was a mixture of views regarding AOD use in CALD communities, although they were mostly negative views. The concept of AOD being ‘taboo’ was frequently referenced by participants, especially in relation to cultural and religious beliefs among many CALD communities. A participant captured this sentiment, stating:

“Oh!” It's like taboo…It's taboo in our culture and religion…Others are just hiding their use because it's a taboo… The problem is they cannot say to others that, “We drink, and we use alcohol, or we use drugs. They cannot say that. They hide to use” (Religious leader, 2)

The above quote reflects the deeply ingrained prohibitions against not only drugs but alcohol within certain communities. In many cultures and religious traditions, alcohol and drug consumption are not just discouraged but outrightly forbidden and viewed as morally or spiritually unacceptable.

A participant noted that when AOD use happens, this is perceived as not normal because a person will only use substances if they are experiencing challenges. However, the community is generally against using substances:

So, what I'm saying is alcohol and drug abuse is not viewed as normal occurrences within our community. So, if, it kind of happens, it's, it's taken as an outlier and it's also viewed as being against the norms; the generally expected norms. Normally, it is associated with either someone having issues, you know, and challenges they've been through, and that kind of thing. And we believe that it's not normal to use alcohol or drugs (Community leader, 8)

In some CALD communities, people who use AOD often experience shame and stigma on three levels: from self, family and community. The shame associated with consuming AOD leads people to deny their use even when they are experiencing harms, therefore making them unlikely to seek help. Shame from the family and community further isolates them which can entrench harms. A community leader referred to this as ‘*cultural embarrassment*’.

Shame doesn't seem like the right word when I say it. In our local language, it's got this deeper sense of shame. It's a cultural embarrassment (Community leader, 6)

For other participants, the stigma experienced also varies depending on the substance in question. Certain substances, like marijuana or cocaine, might be perceived as less serious or stigmatised compared to other drugs like heroin or methamphetamine (‘ice’). This hierarchy of stigma affects how individuals are treated within their communities. Using marijuana or cocaine might be viewed with some level of disapproval, but it doesn't carry the same intense stigma as injecting heroin, which is often seen as far more severe and shameful in Muslim communities:

It is very stigmatized because people have to live up to expectations of their family and their religion…when someone starts using, it's always very secretive and there's a lot of denial behind it… I think also, depending on the substance, the perception of community may change a little bit. You know, if someone's smoking marijuana versus someone injecting heroin… if there's needles involved, it's a much different ball game versus if it's smoking a cigarette. Like it's a little bit less stigmatised. So, very similarly with cocaine: it's a little bit less stigmatised than having ice… someone would say, “Oh, I only do cocaine.” So, that to me tells me they are minimising it. Then they hide using ice or the other substances (Community leader, 1)

Furthermore, the stigma attached to substance use can make it difficult for individuals to ‘*own the problem*’, as noted by a religious leader. Instead, AOD use becomes something to be concealed, resulting in a lack of discussion and awareness. This can perpetuate a cycle of secrecy, where individuals are discouraged from seeking treatment, fearing judgement from their families or communities. The pressure to maintain appearances and uphold cultural expectations can further entrench feelings of shame, making recovery even more challenging.

I mean it is impermissible for us as Muslims to drink alcohol… And also, by extension, any drug because it has the same effect as alcohol; it removes the intellect. So, therefore that plays a factor regarding how people approach the matter. Also, because of this strong impermissibility, there is a very deep sense of shame if someone is found to be an abuser of alcohol and of drugs. Therefore, it's always tucked underneath the rug, you know. We can't speak about it or address it (Religious leader, 7)

The stigma and shame also stem from religious prohibitions against AOD use within certain communities. The strong moral and spiritual frameworks provided by religious texts, like the Quran and Bible, often position substances like AOD as inherently harmful, with the potential benefits far outweighed by the negative consequences.

In the Quran, it talks about intoxicants and gambling, there's benefit and there's harm. But the harm outweighs the benefit. So, stay away from it (Community leader, 1)

What I can say is that people from my country are broadly religious. So, and most of, most of us are Christians. And then we have a bit of Muslims. But you find that that general perception of alcohol and drug abuse from a religious point of view is not taken lightly, and it's not something which is celebrated (Community leader, 4)

These religious teachings act as guiding principles for behaviour, creating a moral and spiritual imperative to avoid AOD use. On one hand, these teachings can be a protective factor, discouraging substance use and at the same time contribute to significant stigma and shame.

#### 4.1.2 Social Dynamics and Access

Although some leaders recognise that alcohol is often used socially or recreationally within their communities—such as during celebrations or special events like birthdays—they also stress the importance of educating people on both the recommended guidelines for safe alcohol consumption and the harmful effects excessive use can cause.

But alcohol, because it's more acceptable, there's more use of alcohol for recreation or celebration…it could be for a birthday…but then sometimes I think what we need is more education about the effects of alcohol; especially, for our people. I always see the bad sides of alcohol (Community leader, 6)

The emphasis on education suggests that in some CALD communities, people may not fully understand the harmful effects of alcohol therefore with targeted education, it may reduce alcohol‐related harm.

The accessibility of alcohol in Australia can be a significant factor influencing increased use among those who have migrated from countries where access to these substances is more restricted. In Australia, alcohol, in particular, is easily accessible and widely accepted as stated by a leader:

But, also, more importantly, is access. I don't think in other parts of the world where many come from might not necessarily have very easy access to alcohol and other drugs, whereas here in Australia, for example, alcohol is readily available. And anyone can buy it, and it's not illegal; it's fine (Religious leader, 7)

For people who come from cultures where AOD use is strictly regulated or tabooed, the ease of access in Australia can increase the likelihood of experimentation or regular use. The normalisation of alcohol consumption can make it harder for people from CALD backgrounds to navigate their relationship with substances, especially when faced with conflicting cultural values or the absence of similar restrictions they were used to in their country of origin.

For some migrants, AOD became a way to cope with the stressors and challenges they face in their new country. Unfortunately, this can lead to a harmful cycle of AOD use and mental health issues, as reliance on AOD to cope can worsen both physical and emotional well‐being in the long run.

Most of the time, you find that migrants are probably struggling to find work or work within their line of professional experience and study, and that kind of thing. So, they find themselves in, in a space of anxiety and frustrations… And, and probably that drags them into, you know, depression and that alcohol and drug use (Community leader, 8)

The above quote shows the importance of providing support systems for migrants to help them navigate the difficulties they face without resorting to harmful coping mechanisms.

### 4.2 Theme 2: Community‐Wide Impact

This theme captures participants' reflections on the collective nature of CALD communities. They often have strong family bonds, tightly knit social groups and shared responsibilities; therefore, the actions of an individual can affect the entire community. Participants described how AOD use disrupts family dynamics, contributes to domestic violence and undermines trust and cohesion within households and communities. These effects extend beyond the individual, destabilising family units and harming broader social relationships, thereby diminishing community wellbeing.

… alcohol was always the contributing factor to the domestic violence in the home (Community leader, 6)

So, the family members, friends, and the people who are around you get really frustrated with the person…it affects the whole community. First, the family and then the relations…the community is affected (Religious leader, 5)

In some instances, the negative impact of AOD on others in the community is a deterrent to other members of the family.

… my sister had a drug issue and it led to a drug‐induced psychosis…watching her experience has actually impacted my children and others not to touch drugs because it can affect your wellbeing quite negatively (Community leader, 6)

Some leaders were concerned AOD use, especially among young people, can negatively impact their education and future opportunities. When student's dropout of school, it affects not only their personal growth but also places a burden on families and limits the potential of the community's next generation:

In our community, we see some younger generation using drugs… they don't go to school, then later dropout of school…they don't have time to study (Religious leader 2)

The social, emotional and economic toll of AOD is felt throughout the community, calling for collective responses to address these challenges.

### 4.3 Theme 3: Enablers to Access and Engagement With AOD Services

This theme highlights the factors that facilitate access to and engagement with AOD treatment services in CALD communities. It includes the availability of cultural wellbeing plans, representation of people of CALD backgrounds in services, and the involvement of religious and community leaders in encouraging treatment.

#### 4.3.1 Culturally Responsive Approaches

This subtheme encapsulates the development of a treatment model which is aligned with the beliefs and values of CALD communities. It also highlights the necessity of recruiting people of CALD backgrounds with the skills in the sector and cultural competency training for existing professionals. Designing a treatment model that is not only culturally sensitive but also adaptable across various groups within the broader CALD communities is important, as noted by a participant, ‘… have like a cultural wellbeing plan’ (Community leader, 1). A one‐size‐fits‐all approach is unlikely to meet the needs of such a diverse population. This requires a thorough understanding of the values, traditions and belief systems specific to each cultural group and a willingness to collaborate with community and religious leaders to ensure relevance and effectiveness.

Come up with a plan to build a treatment model for CALD communities and within the CALD communities as well that's also relevant to other branches within the CALD community (Religious leader, 7)

In addition to having a model, a community leader emphasises that frontline workers who share the same cultural background can better understand and address the specific challenges faced by CALD individuals.

I would encourage that there are more culturally appropriate people trained or skilled to deliver the program to the people… The workers that are in the treatment centre are from a CALD background, which I think it's really important that people can connect well with them, and they feel immediately connected (Community leader 6)

Another aspect of being culturally aware is for service providers to have some knowledge about clients' cultural background if possible. A community leader reported that services need to ‘identify the high numbers and then identify what's the primary values in that culture. And, when you identify someone from that culture coming to your service, you provide those value‐based treatment’ (Community leader, 1). When service providers take the time to learn about the cultural values, beliefs and practices of their clients, they are better equipped to offer treatment that resonates with the client's values.

I think, from our community's perspective, people value returning to faith and realigning their sense of values. And, if we're [the Services] not accepting that and we don't align their sense of values with our treatment, it's planning to fail. You've got to align their values with treatment and then our treatment will be a lot more effective… (Community leader, 1)

… at least in my experience, when I speak to those who are struggling with alcohol and drugs, they always say that faith is a major factor to help them, overcome this challenge (Religious leader, 7)

Understanding the above perspective is important because cultural values often influence how people perceive and respond to health interventions, including their attitudes toward AOD use and recovery processes.

#### 4.3.2 Involving Families

The role of the family is important in the treatment process and essential to foster support. It is particularly relevant in CALD communities, where family sometimes plays a central role in decision‐making and support structures. The community leaders' recognition of the family's role reflects a culturally sensitive approach to treatment, where family involvement can enhance both the emotional and practical aspects of recovery. In the event that family dynamics might be strained or where stigma within the family could hinder recovery, this needs to be managed well.

Should family be involved? Definitely…it helps the treatment to succeed. It can be very tiring and frustrating, and painful, and hurtful for the person that's going through that treatment. It can be quite an emotional journey…I believe that's very important that not only that the family members but the community to support for it to be a success (Community leader, 6)

Another participant acknowledged that if the individual prefers the family to be involved, then the opportunity should be provided. This points to the importance of respecting client autonomy, ensuring that treatment decisions align with their preferences.

Yeah, if they know about the issue, they 100 per cent should be involved if there isn't any concern of confidentiality, then I think then there can definitely be a lot of support (Community leader, 1)

I think it depends on the family itself. So, if the family is supportive, understands and is willing to learn, then I think they should be in the journey from the get‐go (Community leader, 7)

The above quote suggests that even though families should be involved, not all families may be automatically helpful in a treatment context as they may even contribute to the problem. It highlights the need for education for families so they can provide more effective support and or receive support themselves.

#### 4.3.3 Safe and Supportive Environments

Providing welcoming spaces where individuals feel comfortable expressing themselves, free from judgement and stigma, can significantly enhance engagement and treatment outcomes.

And then we need community consultations and co‐design around what the treatment centre needs to look like as soon as someone walks in. So, the look and the feel of the centre. So, when you walk in, what do you see and how does it make you feel? This will then determine whether you would feel comfortable to engage or not (Community leader, 1)

The community leader highlights the importance of designing treatment environments that resonate with the cultural and emotional needs of the people they serve. It emphasises the need for community consultation in creating spaces that are visually and emotionally welcoming. This includes considerations such as aesthetics, colour schemes, and overall ambiance, which can greatly influence a person's comfort and willingness to engage with the service.

### 4.4 Theme 4: Barriers to Access and Engagement With AOD Services

This theme addresses the obstacles that prevent individuals from seeking or completing AOD treatment. These barriers include stigma and shame, communication and awareness and cultural competence.

#### 4.4.1 Stigma and Shame

Stigma and shame play a significant role in preventing people from seeking help for AOD problems in CALD communities. This may be due to fear of being judged by others in the community. Because of this stigma, people are more likely to conceal their drug use and avoid seeking help, even when they are experiencing harm. The shame they feel can make it difficult to admit to themselves or others that they have a problem, leading them to suffer in silence.

I think because substances are seen as unacceptable in our community, there is a little bit of stigma about it. So, there is shame that's attached to it. So, yes, people will not openly come out and say, “Yes, I'm actually struggling with this“ (Community leader, 8)

This reluctance to seek support means that their problems may worsen over time, as they miss opportunities for early intervention or treatment. To remove these barriers, it's crucial to address the stigma surrounding substance use within these communities and create supportive, non‐judgmental environments.

#### 4.4.2 Communication and Awareness

Language barrier can impede understanding and engagement with treatment services, especially for people who have limited proficiency in English. Service providers without interpreter services are less likely to be engaged by some CALD communities, even if people recognise the need for treatment.

If they have a drug treatment, a drug problem, better they go to get treatment soon as possible. But, as I said, one barrier is language… the first thing is their English is not good enough to participate in activities. The second thing is their English is very limited. So, that's a barrier for them (Community leader, 4)

The language barrier affects multiple aspects of the treatment process. People may not be able to fully participate in treatment activities, such as group discussions or educational sessions, and can lead to feelings of exclusion and frustration making it harder for them to engage with treatment. Secondly, those with limited English skills may struggle to navigate the system to access these services in the first place.

Furthermore, there appears to be difficulty for communities in knowing what services are available, let alone accessing them. This gap in information extends beyond just awareness of services; it also includes a lack of understanding about the nature of addictions.

There isn't a lot of support. There isn't a lot of information around how to access support; what services are available in the mainstream. Also, just lack of information around understanding the nature of substance‐use addictions (Community leader, 1)

The above quote suggests that in many CALD communities, there may not be enough outreach or education about the types of support systems available, such as rehabilitation centres, counselling services or harm reduction programme. Without accessible information, individuals may not recognise the severity of substance use or know where to turn for help which delays help‐seeking.

#### 4.4.3 Cultural Competence

The lack of culturally sensitive services can lead to disengagement with services for people of CALD backgrounds, especially those from specific religious communities like Muslims, in engaging with treatment programme. The quote from the religious leader underscores the importance of providing services that align with the cultural and religious beliefs of these communities.

… if we have a model that's specific for Muslims and those who are in the CALD community or Arab community as well, then no doubt the uptake of treatment would be at least tenfold. And the reason being is because the comfort that one finds behind an organisation that's faith‐based with Islam is second to none. It's a recurring theme that we hear often, so many times, that they want to connect with their faith but they can't. Why? Because they're at a facility where perhaps it doesn't offer it or there may be activities that they might have to get involved in where it may be contrary to their faith and they don't know whether they can do it or they can't do it (Religious leader, 7)

One of the sticking points for us as Muslims, even for, for Imams, is, okay, even if we are to refer these, these people who need help, where are we going to refer them to where the service is actually tailored for them as part of a CALD community and, more specifically, a Muslim community? There's nothing with the exception of one program that I know of which is running (Religious leader, 7)

The above quote illustrates how the absence of culturally sensitive services can create a barrier to treatment. People from CALD backgrounds may avoid seeking help altogether or feel uncomfortable staying in programme that don't cater to their specific needs, values and beliefs.

There are some cultural resistances from some CALD communities toward harm reduction strategies, specifically when it comes to AOD treatment. This resistance can be rooted in cultural beliefs or a lack of understanding about what harm minimisation entails. For example, many parents might struggle with the idea that relapse is part of the recovery process. This misunderstanding can lead to the rejection of strategies like harm minimisation, which focus on reducing the negative effects of drug use rather than an all‐or‐nothing approach to quitting.

I think the fact that, so some parents don't like the idea to say relapse is a possibility. So they have an issue with that concept of harm minimisation…Sometimes, it depends if they have an understanding of what medications can do to prevent harm, they don't want any medication. Because it's just replacing their addiction with something else. But, if you're on opioids and you're on heavy opioid use, and you can have a medicated‐assisted detox, and some sort of intervention for the next month or two, to help ease the initiation of your recovery, I agree (Community leader, 1)

When treatment providers do not examine how cultural beliefs and misconceptions about harm reduction may contribute to a rejection of harm reduction strategies, people from these communities may be less likely to seek or accept help, further isolating them from the essential support they need to recover. It is important to understand these dynamics when working with CALD communities therefore educating families, religious and community leaders about medication‐assisted programme and harm reduction strategies can make harm minimization more acceptable and accessible.

## 5 Discussion

This study aimed to explore the perceptions of religious and community leaders toward AOD use, harms and treatment access in CALD communities. Four main interconnected themes were identified: cultural and contextual factors; community‐wide impact; treatment enablers; and treatment barriers. Cultural and contextual factors shape community norms and expectations, which in turn influence the collective responses captured in the theme of community‐wide impact. These shared values and perceptions can either facilitate or hinder access to treatment, as seen in the themes of treatment enablers and barriers. The findings reveal that AOD use remains heavily stigmatised and tabooed within some CALD communities, with many leaders sharing this belief. AOD use is often seen as morally wrong and contrary to cultural and religious beliefs. Due to taboos that are dominant in some CALD communities, they may be reluctant to admit AOD use [28]. Similar findings have been reported in studies conducted among CALD communities in the United Kingdom and Australia, where abstinence from AOD is strongly encouraged, and individuals who engage in substance use face shame and bring dishonour to their families [29, 30]. In many CALD communities, family reputation is prioritised over individual actions, so the behaviour of one person affects not only themselves but also their entire family and broader community. As a result, stigma and shame surrounding AOD use often lead to secrecy, with individuals hiding their drug use, which can worsen the associated harms. Additionally, negative perceptions around injecting drug use are particularly strong in CALD communities. This is consistent with qualitative research among African migrants and refugee youth in Melbourne, who reported cultural beliefs that strongly oppose injecting drug use [7].

### 5.1 Acculturation Pressures

In some instances, there is a dissonance between cultural and religious beliefs toward alcohol use of some CALD communities against the liberal drinking culture in Australia. Findings from this study showed that alcohol was more accessible in Australia than in their country of origin, hence likely to influence excessive use. Studies among migrants in Australia have reported similar findings where newly arrived migrants, in an attempt to acculturate in Australia, may experience pressure to drink to conform [31, 32, 33]. This can lead to contentions between maintaining cultural identity and adapting to the norms of the host country. Such tension may result in feelings of guilt, shame and conflict within families or communities, particularly for those whose religious or cultural practices discourage alcohol consumption. Ultimately, these pressures can increase the risk of alcohol‐related harms and are a barrier for individuals to seek help, especially when facing stigma or judgement from their community [3].

### 5.2 Systemic Barriers

There are systemic barriers that migrants face, including access to healthcare, employment and other social services [34, 35, 36]. In this study, systemic barriers such as the lack of recognition of migrants' overseas qualifications emerged as a significant factor influencing AOD use. Difficulties experienced by migrants when attempting to secure employment, particularly in roles that correspond to their professional experience or educational background, were highlighted. Other studies have reported risk factors for AOD use among people of CALD background, including unemployment, a lack of meaningful work and limited education [6]. The stress of underemployment or unemployment can take a profound psychological toll. This aligns with prior research showing that prolonged unemployment or underemployment among migrants can significantly increase the risk of mental health disorders [37, 38]. AOD is therefore a mechanism to manage these overwhelming emotions. This study underscores the pressing need for targeted support services that not only address AOD use but also provide holistic interventions to manage the mental health and social integration for migrants.

### 5.3 Culturally Appropriate Treatment

Providing culturally appropriate treatment is crucial for enhancing treatment engagement among CALD groups. Evidence shows that individuals from CALD backgrounds are less likely to access or engage in AOD treatment due to several factors, including the lack of culturally tailored services and inadequate definitions of who qualifies as CALD [3, 23]. An example of a culturally specific service relevant to this study setting is the Odyssey House Multicultural Program, which also collaborated on this research [39]. Based in Western and South‐Western Sydney, the programme provides tailored AOD support for people from CALD backgrounds. Staff members speak over 20 languages and deliver culturally appropriate interventions that reflect the diverse needs of the communities they serve. Providing culturally responsive care is one of the principles for effective AOD treatment as recommended by the National Framework for Alcohol, Tobacco and other Drug Treatment [10].

For treatment services to be effective, they must also demonstrate cultural competence and ensure adequate representation of CALD individuals within the workforce. Incorporating cultural and religious values and beliefs within traditional treatment plans further enhances individuals' healing. Learning about the cultural values also helps the service providers to avoid cultural misunderstandings or biases that may otherwise hinder the therapeutic relationship. This approach fosters better engagement and adherence to treatment because clients feel that their unique cultural needs are acknowledged, ultimately leading to more successful treatment outcomes. The National Practice Standards specifically state that mental health workers should liaise or work collaboratively with CALD care partners such as religious and spiritual leaders, traditional healers and community‐based organisations where appropriate [40]. By fostering these collaborations, treatment services can create a more inclusive and supportive environment that better meets the needs of CALD communities.

### 5.4 Information and Education

Participants in this study highlighted a gap in the availability of information and education on AOD use, as well as safe alcohol consumption guidelines within CALD communities. Similar findings have been reported in CALD populations in Victoria [3, 6]. There is a need for educational resources and health promotion campaigns within these communities, ideally developed in collaboration with community and religious leaders to ensure that the messaging is culturally relevant [9]. Involving CALD leaders in such initiatives can foster greater trust in AOD services and reduce fears surrounding the disclosure of AOD use, which some individuals may associate with risks to their visa status or residency applications, as well as potential discrimination [41]. Education should also target the reduction of stigma and shame associated with AOD use, while increasing awareness of available services and how to access them [3, 9]. Addressing stigma in CALD communities requires culturally appropriate and community‐informed approaches. Effective strategies include engaging community members and organisations in the co‐design of health programme and messaging, ensuring that interventions are contextually relevant and respectful of cultural values [42, 43, 44]. Other strategies such as culturally tailored interventions, working with bicultural workers [45], using family‐based interventions [45], peer networks and collaborating with religious and community leaders all play a pivotal role in shaping cultural norms and perspectives [45]. Since many CALD communities possess cultural and religious beliefs that act as protective factors against AOD use, building on these values in prevention and treatment efforts may improve engagement with services [46]. Harm reduction is another concept that is unfamiliar to many CALD communities. Therefore, education on harm reduction strategies is essential and should ideally be led by community members themselves [6].

Furthermore, for some CALD groups, seeking help outside of the family is uncommon, as the family unit is typically the primary source of social support [46]. Thus, efforts should also focus on educating family members about AOD issues, equipping them with the knowledge needed to support their loved ones. Engaging bicultural workers in these educational efforts and providing culturally competent training to service providers will further enhance the effectiveness of these interventions [3, 46].

### 5.5 Implications for Policy and Practice

There remains limited knowledge on the prevalence and patterns of AOD use within CALD communities in Australia. This lack of data presents a significant barrier to the development of effective and culturally responsive AOD services. Targeted funding is needed to support further research in this area, particularly to generate robust evidence and inform the design of tailored treatment frameworks that address the unique needs of diverse CALD populations. Effective responses must be co‐designed in partnership with CALD communities, including religious and community leaders. Such partnerships can enhance trust, increase the uptake of services and support culturally appropriate health promotion campaigns aimed at reducing stigma and normalising help‐seeking behaviours. In addition, there is a pressing need to invest in training and education programme for religious and community leaders. These programme should focus on building understanding of AOD‐related issues, the impact of stigma and the critical role leaders can play in supporting community members to access care.

### 5.6 Strengths and Limitations

Although the qualitative exploratory approach offers in‐depth data gathering and analysis, it is associated with difficulties in generalising the findings of the study beyond sampling boundaries due to its subjective nature of interpretations. Thus, the current findings of the study may not be representative of other communities or cultural settings outside those included in the study. Nevertheless, the findings of the present study can offer direction for future studies in other communities and settings. Another limitation of the study is the use of purposive sampling technique. Although purposive sampling helped the researchers to identify participants with detailed knowledge of AOD use and treatment within their communities, the findings may be limited by data being gathered from available and willing participants.

## 6 Conclusions

AOD problems in CALD communities are complex and multifaceted. Our findings suggest that culturally sensitive interventions and services are required, and addressing systemic level barriers is crucial to improving access to treatment and support. This can involve implementing policy changes, increasing funding for treatment programme and engaging in community partnerships to increase education and awareness campaigns aimed at reducing stigma and promoting access to healthcare services.

## Conflicts of Interest

The authors declare no conflicts of interest.

## Data Availability

The data that support the findings of this study are available from the corresponding author upon reasonable request.
